# How to Prevent Drowning

**Published:** 1881-11

**Authors:** 


### HOW TO PREVENT DROWNING.

A writer in "Nature" says : I wish to know how drowning might, under ordinary circumstances, be avoided even in the case of persons otherwise wholly ignorant of what is called the art of swimming. The numerous frightful casualties render every working suggestion of importance, and that which I here offer I venture to think is entirely available.

When one of the inferior animals takes the water, falls,or is thrown in, it instantly begins to walk as it does when out of the water. But when a man who cannot "swim" falls into the water, he makes a few spasmodic struggles, throws up his arms, and drowns. The brute,on the other hand, treads water, remains on the surface and is virtually insubmergible. In order, then, to escape drowning, it is only necessary to do as the brute does, and that is to tread or walk in the water. The brute has no advantage in regard of his relative weight,in respect of the water, over man, and yet the man perishes while the brute lives. Nevertheless, any man, any woman, and child, who can walk on the land may also walk in the water just as readily as the animal does, if only he will, and that without any prior instruction or drilling whatever. Throw a dog into the water and he treads or walks instantly, and there is no imaginable reason why a human being under like circumstances should not do as the dog does.

The brute in the water continues to go on all fours, and the man who wishes to save his life and cannot otherwise swim must do so too, striking alternately, one, two, one, two, but without hurry or precipitation, with hand and foot exactly as the brute does. Whether he be provided with paw or hoof, the brute swims with the greatest ease and buoyancy. The human being, if he will, can do so too, with the further immense advantage of having a paddle-formed hand, and of being able to rest himself when tired by floating, a thing of which the animal has no conception.

The loss of life from shipwreck, boating, bathing, skating, fishing and accidental immersion is so disastrously great that every feasible procedure calculated to avert it ought to be had recourse to. People will not consent to wear life preservers, but if they only knew that in their own limbs, properly used, they possessed the most efficient life-preservers, they would most likely avail themselves of them. The printed injunction should be pasted on all boat-houses, on every boat, at every bathing place, and in every school. "Tread water when you find yourself out of your depth" and "Float when you are tired." Every one, of whatever age or sex, or however encumbered with clothing, might tread water with at least as much facility, even in a breaking sea, as a four-footed animal does. The Indians on the Missouri river, when they have occasion to traverse that impetuous stream, invariably tread water just as the dog treads it. Young persons of both sexes, of the natives of Joanna, an island on the coast of Madagascar, walk the water, carry fruit and vegetables to ships becalmed, or it may be lying to, in the offing miles away. Some Croomen, whose canoe upset before my eyes in the seaway on the coast of Africa, walked the water to the safekeeping of their lives, with the utmost facility, and I witnessed negro children on other occasions doing so at very tender age. At Madras, watching their opportunity, messengers with letters secured in an oilskin cap plunge into the boiling surf, and make their way, treading the water, to the vessels outside, through a sea in which an ordinary European boat will not live. At the Cape of Good Hope men used to proceed to the vessels in the offing through the mountain billows, treading the water as they went with the utmost security. And yet here on our own shores and amid smooth waters, men, women and children, perish like flies annually, when a little properly directed effort—treading the water as I have said—would happily suffice to rescue them, every one.

